# Effect of Unanticipated Tasks on Side-Cutting Stability of Lower Extremity with Patellofemoral Pain Syndrome

**DOI:** 10.3390/s24196427

**Published:** 2024-10-04

**Authors:** Yiwen Ma, Wenjing Quan, Xuting Wang, Julien S. Baker, Zixiang Gao, Yaodong Gu

**Affiliations:** 1 Faculty of Sports Science, Ningbo University, Ningbo 315211, China; 2311040015@nbu.edu.cn (Y.M.); 15536948303@163.com (X.W.); 2 Department of Sport and Physical Education, Hong Kong Baptist University, Hong Kong, China; jsbaker@hkbu.edu.hk; 3 Human Performance Laboratory, Faculty of Kinesiology, University of Calgary, Calgary, AB 403, Canada; gaozixiang0111@outlook.com

**Keywords:** patellofemoral pain syndrome, biomechanics, unanticipated side-cutting, knee joint mechanics

## Abstract

Background: Patellofemoral pain syndrome (PFPS) is one of the most common causes of anterior knee pain encountered in the outpatient setting. The purpose of this study was to compare the lower limb biomechanical differences during anticipated and unanticipated side-cutting in athletes with PFPS. Methods: Fifteen male basketball players diagnosed with PFPS were enrolled in the study. Participants executed both anticipated and unanticipated 45-degree side-cutting tasks. Motion analysis systems, force plates, and electromyography (EMG) were used to assess the lower limb joint angles, joint moments, joint stiffness, and patellofemoral joint contact forces. Analyzed biomechanical data were used to compare the differences between the two circumstances. Results: Unanticipated side-cutting resulted in significantly increased ankle plantarflexion and dorsiflexion angles, knee abduction and internal rotation angles, and hip abduction angles, as well as heightened knee adduction moments. Additionally, patellofemoral joint contact forces and stress increased, while contact area decreased during unanticipated tasks. Conclusions: Unanticipated movement raises the demands for joint stability and neuromuscular control, increasing injury risks in athletes with PFPS. These findings have practical implications for developing targeted rehabilitation programs and injury prevention strategies.

## 1. Introduction

Knee pain is a prevalent musculoskeletal ailment, affecting as many as 54% of athletes annually [1]. Knee injuries are prevalent, and patellofemoral pain syndrome (PFPS) is a frequently occurring ailment [2]. PFPS is defined by discomfort in the front of the knee, commonly worsened by activities such as jogging, jumping, and ascending stairs [3,4,5]. Over 50% of individuals with PFPS may continue to experience knee pain and impaired function for 5–8 years [6]. Additionally, initial symptoms of anterior knee discomfort might progress and lead to the development of patellofemoral arthritis [7]. The primary causes of PFPS include abnormal patellar tracking, muscle imbalances, and poor lower limb alignment [8]. During common movements like a cutting maneuver, these biomechanical issues can lead to increased stress on the knee joint, further aggravating PFPS [9]. Understanding the etiology and biomechanical impact of such activities on the knee is crucial for effective diagnosis and treatment.

Side-cutting movements typically result in significant biomechanical changes in the knee joint, such as increased joint loading, altered kinematics, and heightened stress on the surrounding musculature and ligaments [10]. During side-cutting movements, the patellofemoral joint (PFJ) experiences considerable stress due to increased knee flexion angles and the high forces produced by the quadriceps. The compressive stress on the PFJ is determined by the combined effect of knee flexion and quadriceps force. The cumulative compressive forces at the PFJ are determined by the interplay between the angle of knee flexion and the force exerted by the quadriceps. To change direction, an athlete must initially decelerate, subsequently turn their body in the new direction, and finally, accelerate in that new direction [11]. During these motions of deceleration and acceleration, which are accompanied by frequent changes in direction, higher moments of knee varus/valgus and decreased knee flexion are recognized as possible risk factors for a knee injury [12,13]. Research indicates that male football players have a significantly higher knee flexion angle during the initial foot contact than female athletes. Additionally, there are notable gender disparities in the varus and valgus angles of the knee joint [14,15]. Elevated knee abduction moments frequently result from the initial contact angle, internal hip rotation, and trunk rotational angles. These factors contribute to increased loading forces on the knee joint. Furthermore, the findings suggest that excessive joint stiffness may increase the high incidence of knee joint injuries [16]. Typically, the laboratory provides prior notice of the intended cutting direction during a test procedure and ensures the participant is adequately prepared. Thus, when compared to testing conducted under anticipated conditions, testing conducted under unanticipated events can more accurately replicate the actual conditions of the competitive environment. Unanticipated conditions refer to situations where the participant lacks prior knowledge of the direction or route of the test action and must rely on visual prediction to make spontaneous reactions. A prior study has found that unanticipated cutting actions are associated with a higher likelihood of damage compared to planned movements. This is because the lack of preparation time in unanticipated cuts leads to less optimal body positioning and muscle activation patterns, increasing the likelihood of injury. Research has shown that unanticipated reductions in movement cause an increase in the knee’s maximum bending, sideways knee movement, and greater angles of the knee bending inward. These factors are related to a higher likelihood of sustaining knee injuries, such as tears in the ACL and PFPS. Previous studies have primarily concentrated on the kinematic and kinetic variables of the knee joint in healthy individuals compared to those with PFPS. However, there is a paucity of research that compares the knee biomechanical parameters during anticipated versus unanticipated movements in individuals with PFPS [17,18,19,20]. As a result, it remains unclear whether anticipated and unanticipated tasks affect knee kinematics in PFPS populations.

Comparing the biomechanical changes during cutting maneuvers between individuals with PFPS and healthy populations reveals significant differences [21]. Previous studies have indicated that sagittal plane knee kinematics, including displacement and peak angles during landing, are crucial factors in determining the magnitude of vertical ground reaction force (GRF). It was surmised that the peak vertical GRF would increase and the time to peak vertical GRF would decrease during unanticipated side-cutting, attributed to reduced lower extremity sagittal plane joint displacements [14,22]. While healthy individuals typically exhibit more controlled and stable knee joint kinematics during cutting, those with PFPS often show altered movement patterns and increased joint stress. Previous studies have investigated unanticipated cutting maneuvers, different cutting angles, and transverse cuttings. This study specifically examined the characteristics that affect completion times of 45° and 90° side-cutting, to identify the factors associated with performance and the loading on the knee joint in the frontal plane [23]. Other research has indicated that alterations in the movement and forces of the lower limbs, resulting from cognitive intervention, may be responsible for the heightened stress experienced by the PFJ [24]. Suda injected hypertonic saline solution (1 mL, 6%) in the knee to induce experimental patellofemoral pain in healthy subjects and found that cognitive interference reduced the subjects’ postural stability, especially in the painful state, which led to increased patellofemoral joint stress [25]. Despite these findings, research focusing specifically on PFPS patients under anticipated and unanticipated conditions remains sparse. Examining how these movements impact these people’s biomechanics of the lower limb and knee joint is essential. Understanding these differences is essential for developing targeted rehabilitation and injury prevention strategies. Studies have laid the groundwork by showing that unanticipated cutting maneuvers significantly impact knee joint mechanics, but similar comprehensive studies in PFPS populations are lacking [9]. While studies have identified key biomechanical alterations in PFPS patients, such as increased patellar dysplasia and altered muscle activation patterns, there is limited data on how these factors vary between anticipated and unanticipated movements. This gap underscores the need for more detailed biomechanical analyses considering the context of movement anticipation.

Weakness in the quadriceps can lead to a more significant lateral displacement of the patella, which in turn causes the patella to move outward, placing joint pressure on the lateral surface, resulting in an excessive Q angle, and subsequently leading to PFPS [24]. The muscle activation results of previous studies show that in the 45° side-cut movement, the quadriceps and gastrocnemius are the muscles that mainly play a role in the side-step cut movement [26]. In addition, the biceps femoris also contributes significantly to the propulsive phase. During unanticipated tasks, the decreased muscle forces of the gastrocnemius, rectus femoris, and vastus medialis muscles indicate a change in neuromuscular strategy to preserve equilibrium and manage rapid shifts in direction [27]. Previous research has demonstrated muscle activation patterns’ complex and joint-specific nature in response to unanticipated movements [8,24,28]. The increased reliance on certain muscles, such as the biceps femoris, and the decreased activation of others, like the rectus femoris and gastrocnemius, suggest that training programs should focus on enhancing the adaptability of the neuromuscular system to improve performance and reduce injury risk in dynamic sports activities [3,4,9,29].

Insufficient research has been conducted on the knee joint in persons afflicted with PFPS. This study aimed to analyze the specific angles, moments, and forces in the joints of the lower limb, as well as the contact forces, areas, and stresses in the PFJ. This was accomplished while observing individuals with PFPS doing an unexpected side-step cutting task. The study also aimed to compare these findings with the data obtained during an anticipated side-step cutting task. We hypothesized that performing an unanticipated side-cutting exercise would result in a reduced contact area in the PFJ, but would also lead to an increased stress force in the PFJ compared to the anticipated side-cutting tasks.

## 2. Materials and Methods

### 2.1. Participants

Before experimenting, the minimum sample size was determined using G*Power (Version 3.1.9.7) to obtain a statistical power of 80% (α = 0.05, effect size = 0.8). This calculation indicated that a sample size of 14 participants was required. Consequently, the study recruited fifteen basketball players from the Faculty of Sport Science diagnosed with PFPS. The participants had the following characteristics: height 179.29 ± 4.57 cm, mass 71.71 ± 5.22 kg, age 20.43 ± 1.90 years, and an Anterior Knee Pain Scale (AKPS) score of 75.43 ± 4.20. The selection criteria for participants with PFPS were based on previous studies and included (1) a pain level of at least 3 cm on the 10 cm Visual Analog Scale (VAS), where 0 represents no pain, and 10 represents the most intense level of pain [30]; (2) an AKPS score ≤ 85; (3) the presence of posterior or peri-patellar pain; (4) the reproduction of posterior or peri-patellar pain during at least two activities, including squatting, stair climbing (ascending/descending), running, single/double-leg jumping, prolonged sitting, and resisted knee extension; (5) the exclusion of other conditions that could cause anterior knee pain, such as tibiofemoral joint pathology [21]; (6) we selectively chose PFPS participants whose left leg was the dominant leg during exercise. Currently, there is a lack of clear diagnostic criteria for PFPS, and the evidence from clinical examinations and imaging assessments is relatively limited, necessitating a reliance on exclusionary diagnosis [31,32]. Prior to the test, all participants provided written informed permission, and the University of Ningbo Research Ethics Board granted approval for the technique (REB20240623).

### 2.2. Sensors

**Figure 1:**
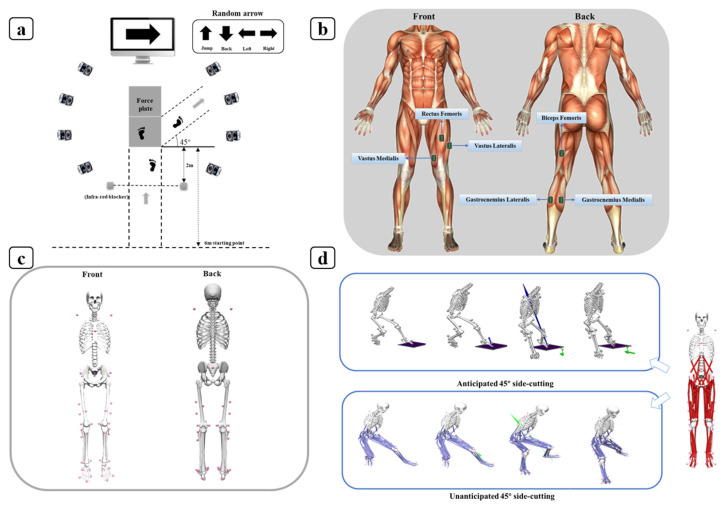
Illustration of experimental scene setup (The grey arrow indicates the direction of the side-cutting) (**a**), EMG acquisition (**b**), reflective point placement (**c**), and schematic diagram of side-cutting action (The blue and green arrows represent the ground reaction forces during a side-cutting) (**d**).

A motion analysis setup featuring eight cameras from Vicon Metrics Ltd. in Oxford, UK, was employed to measure lower limb kinematic parameters at a frequency of 200 Hz [33]. Simultaneously, ground reaction force data were collected using a Kistler Type 9281 B force plate from Kistler Instrument AG in Winterthur, Switzerland, sampling at 1000 Hz throughout the side-cutting activity [34]. A custom timing-gate program was employed to measure the velocity of the approach at the initial, second, and third timing gates and determine the cutting direction for the anticipation condition. The approach speed was constrained to a range of 4.0 to 5.5 m/s, identified as the optimal pace for participants to perform the task effectively based on pilot testing. An infrared blocker was positioned 2 m from the force plate. During the unanticipated condition, when the participant encountered the infrared blocker, it transmitted a signal that prompted the computer to execute the random arrow program (Figure 1a). A display randomly illuminated an arrow, signaling the participant to either side-cut or perform another task [35]. This display provided a randomized visual cue for the direction of the side-cutting. To standardize the direction of the side-cutting, tape was placed on the floor at a 45-degree angle. Participants were instructed to follow the random arrow directions of the turn signal and execute the relevant change in direction. This procedure was designed to test the unanticipated hypothesis by observing the randomly executed side-cutting actions utilizing a 16-channel wireless electromyography (EMG) system with a sampling rate of 1000 Hz (Delsys, Boston, MA, USA) [36]. It is suggested that the movements use many muscles, including the rectus femoris (RF), biceps femoris (BF), vastus medialis (VM), vastus lateralis (VL), gastrocnemius medialis (GM), gastrocnemius lateralis (GL), and others, in muscular exercises (Figure 1b).

### 2.3. Procedures

Before data collection, all participants were briefed on the experimental collection procedure and engaged in a 5-min warm-up to prevent injury. They were required to wear a uniform consisting of tight-fitting T-shirts and pants. To eliminate any potential influence of footwear on the experimental data, we provided participants with standardized footwear. Using double-sided and stretchable tape, 38 reflective markers (14 mm) were secured to their lower limbs following the Opensim 2392 model marker guidelines (Figure 1c). Markers were precisely positioned on the torso (left and right acromion), pelvis (anterior and posterior superior iliac spines), tibia (medial and lateral sides of the knee and ankle on both legs), and foot (from the calcaneus to the toe tip) [37].

Every participant was obligated to sprint five meters and execute a 45° cutting maneuver, while maintaining a sprint speed of 4.0 ± 0.5 m/s. In addition, during the data collection phase of this experiment, all participants were required to perform both anticipated and unanticipated movements during the side-cutting task [33]. Previous studies defined the anticipated task as one in which the participant was provided with prior information regarding the direction (right) before beginning the task [17]. Conversely, the unanticipated task involved participants being instructed to respond and move in the left, right, upward, or downward direction in accordance with an arrow displayed on a laptop screen [38]. The arrow appeared arbitrarily when the participant encountered the infra-red blocker (Figure 1d).

Participants completed four rightward-cutting trials, encompassing both anticipated and unanticipated tasks. Following a side-cutting maneuver, it was necessary for participants to promptly move toward the force platform, placing their left foot in the center of the platform to record data accurately upon disengagement from the ground. The tasks were randomized by direction and type to control for order effects and fatigue. Participants initiated maximum acceleration from the starting position and sprinted six meters toward a force plate, subsequently changing direction to the right and continuing to accelerate. Before the trials, participants were directed to complete a five-minute jogging warm-up. During the data-gathering process, they wore standard athletic shorts and footwear. Before testing, they were introduced to the experimental procedures. Each of the four successful trials involved cutting at a 45° angle to the right, incorporating both anticipated and unanticipated tasks, with each trial followed by a three-minute rest period.

### 2.4. Data Processing

**Figure 2:**
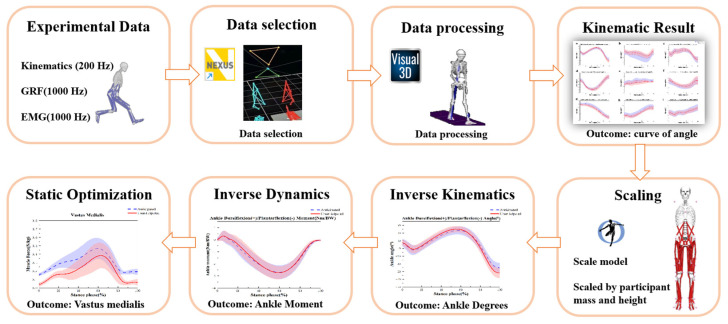
Data collection and processing.

Figure 2 depicts the process of gathering and evaluating data. The study recorded marker trajectories in three dimensions using an 8-camera motion capture system with a sampling rate of 200 Hz. Additionally, ground reaction force data were gathered using Kistler force plates, with a sampling rate of 1000 Hz [36]. The data selection process used Vicon Nexus (version 1.8.5A, Vicon Metrics Ltd., Oxford, UK) software to label and export C3D files. The three-dimensional motion during the stance phase of the side-cutting task was analyzed in this study. The data from the left leg’s ground response force were used for analysis. The data were then exported in C3D file format to Visual 3D software (version 2020, C-Motion Inc., Germantown, MD, USA) for calculating joint kinematics and kinetics parameters during 45° side-cutting. A fourth-order Butterworth low-pass filter with cutting frequencies of 20 Hz and 50 Hz, respectively, was used to handle the kinematics and kinetic data [39]. The Opensim 2392 musculoskeletal model was used in this study. The established process was followed for musculoskeletal modelling [40,41,42]. An initial step of “scaling” was performed to obtain a model with adjusted anthropometric fit. Subsequently, “inverse kinematics” was employed to ascertain the knee flexion angle that minimizes positioning discrepancies between the experimenter and virtual model markers. In Opensim 4.2, a “static optimization” technique was employed to compute muscle activations and forces for the side-cutting movement. This technique aims to minimize the total of squared muscle activations (Figure 2). Maximum voluntary contraction (MVC) of the muscle was performed following a previously established protocol to normalize muscle activity (0–100%) [43].

**Figure 3:**
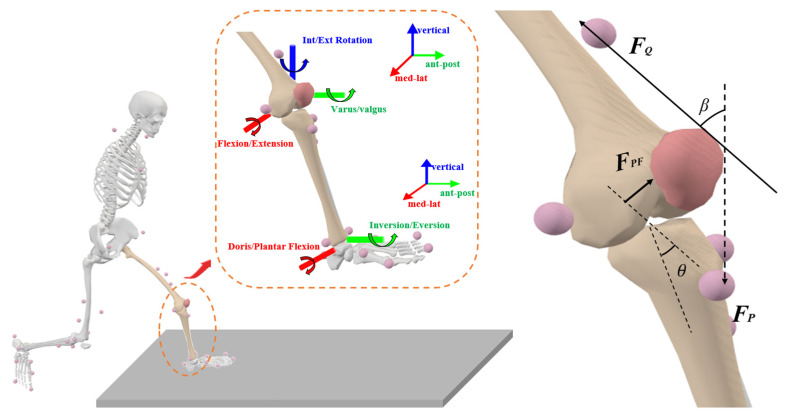
Illustration of the knee and patella parameters. The dotted line shows an enlarged schematic diagram of the patellofemoral joint. Blue represents internal and external rotation of the joint, red represents joint flexion and extension, and green represents joint inversion and eversion.

In addition, the inverse dynamics approach was employed to ascertain the joint moment (Figure 3). This research also assessed the rigidity of the hip, knee, and ankle joints during the stance phase of side-cutting [44]. The formula for determining joint stiffness is as follows:

(1) $$\mathrm{Joint}\;\mathrm{Stiffness}=\frac{{∆M}_{joint}}{{∆\theta }_{joint}}$$

${∆M}_{joint}$ refers to the alteration in joint moment during the side-cutting stance phase, whereas ${∆\theta }_{joint}$ represents a variation in joint angle during the same period.

The knee extension moment ($M_{EXT}$), and knee flexion angle (θ) obtained from biomechanical testing were used to compute the patellofemoral joint contact force ($F_{PF}$), contact area ($S_{PFCA}$), and stress force ($P_{PFJS}$). The units of these calculations are N, mm2, and MPa, respectively [45,46].

(2) $$F_{Q}\left(\theta_{i}\right)=\frac{M_{EXT}\left(\theta_{i}\right)}{L_{A}\left(\theta_{i}\right)}$$

The quadriceps muscle force (N) is represented by $F_{Q}$, the knee extension angle (cm) by $M_{EXT}$, and the knee flexion and extension angle (°) by $\theta_{i}$ of the i-th frame. Additionally, this study also presupposes that the net knee moment in the sagittal plane when cutting corresponds to the knee extension moment when it is positive, that is

(3) $$M_{EXT}=M_{NET}$$

The formula represents the net moment of the knee joint in the sagittal plane as $M_{NET}$, measured in N·m. The effective muscle moment arm of the quadriceps femoris depends on the knee joint angle θ (°) of the knee joint in the sagittal plane,

(4) $$L_{A}=\left\{\begin{array}{c} 0.036\theta +3.0\left(0{}^{\circ}\leq \theta \leq 30{}^{\circ}\right)\\ -0.043\theta +5.4\left(30{}^{\circ}\leq \theta \leq 60{}^{\circ}\right)\\ -0.027\theta +4.3\left(60{}^{\circ}\leq \theta \leq 90{}^{\circ}\right)\\ 2.0\left(90{}^{\circ}\leq \theta \right) \end{array}\right.$$

The formula for calculating patellofemoral joint force is as follows:

(5) $$F_{PF}=2F_{Q}\sin\left(\frac{\beta }{2}\right)$$

where:

(6) β=30.46+0.53(θ)

The formula uses $F_{PF}$(N) to represent the PFJF and β(°) to denote the angle between the quadriceps muscle line and the tension in the patellar ligament. The calculation of patellofemoral joint stress includes the contact area between the patella and the femur (mm^2^) and is a function of the sagittal knee angle θ, measured in degrees (°);

(7) $$S_{PFCA}\left(\theta_{i}\right)=\left(0.0781\times {\theta_{i}}^{2}+0.6763\times \theta_{i}+151.75\right)$$

$S_{PFCA}\left(\theta_{i}\right)$ is the contact area between the patella and the femur (mm^2^), based on which the patellofemoral joint stress is finally obtained;

(8) $$P_{PFJS}\left(\theta_{i}\right)=\frac{F_{PF}\left(\theta_{i}\right)}{S_{PFCA}\left(\theta_{i}\right)}$$

In the formula, $P_{PFJS}$ is PFJS (MPa).

### 2.5. Statistical Analysis

We standardized the data by considering the time of each standing phase, which comprises 101 data points. The data were assessed for normalcy using a Shapiro–Wilk test. An analysis was performed using SPSS statistical software (version 23.0, SPSS Inc., Chicago, IL, USA) to compare the predicted and unexpected tasks. A *p*-value of 0.05 or below was statistically significant.

## 3. Results

### 3.1. Joint Kinematics

**Figure 4:**
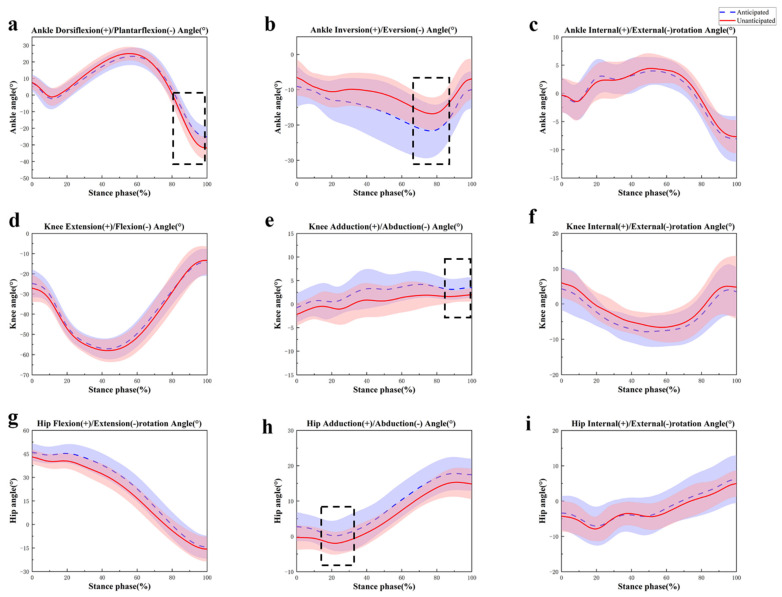
Illustration of the anticipated and unanticipated lower limb results shows the statistical parametric mapping outputs for the angle of the sagittal ankle (**a**); frontal ankle (**b**); transverse ankle (**c**); sagittal knee (**d**); frontal knee (**e**); transverse knee (**f**); sagittal hip (**g**); frontal hip (**h**); transverse hip (**i**) during 45-degree side-cutting. Note: The dashed box index indicates a significant difference between the two side-cutting tasks with *p* < 0.05.

Table 1 presents the changes in peak joint angles observed during anticipated and unanticipated 45° side-cutting. For the ankle, the unanticipated condition resulted in a significant increase in peak ankle dorsiflexion (*p* = 0.036) and plantarflexion (*p* < 0.001) compared to the anticipated condition (Figure 4a). Additionally, the peak inversion (*p* = 0.003) and eversion (*p* < 0.001) angles significantly decreased (Figure 4b). The ankle joint range of motion (ROM) in the sagittal plane was also significantly larger than anticipated (*p* < 0.001). However, the ankle ROM in the transverse plane was lower than anticipated (*p* = 0.008). No significant differences were found in ankle internal and external rotation angles, and there was no significant difference in ankle joint ROM in the frontal plane, Table 2 shows the joint range of motion angel during intended and unintended movements. (Figure 4c).

In the case of the knee, peak knee abduction (*p* < 0.001) and knee internal rotation (*p* = 0.03) during the unanticipated 45° side-cutting were significantly increased compared to the anticipated condition (Figure 4e,f). Conversely, the peak knee adduction angle was significantly lower than anticipated (*p* < 0.001). There were no significant differences in peak knee flexion angle between the anticipated and unanticipated conditions, nor were there significant differences in knee joint ROM across the sagittal, transverse, and frontal planes (Figure 4d).

For the hip, unanticipated tasks led to a significant reduction in peak hip adduction (*p* < 0.001) and hip abduction (*p* = 0.005), while the hip joint ROM in the sagittal plane (Figure 4h) was also significantly reduced (*p* = 0.023). No significant differences were observed between anticipated and unanticipated conditions in peak hip flexion angle, internal rotation, and external rotation (Figure 4g,i).

As shown in Table 3, unanticipated tasks resulted in lower ankle initial foot contact angles (*p* = 0.011) in the transverse plane. The sagittal and frontal planes also have lower hip initial foot contact angles. However, the unanticipated task resulted in a higher knee initial contact angle in the transverse plane. There were no significant differences in the initial foot contact angles of the ankle and knee joint on the sagittal and coronal planes. The hip initial contact angle in the frontal plane was also not different (Table 3).

### 3.2. Joint Kinetics

**Figure 5:**
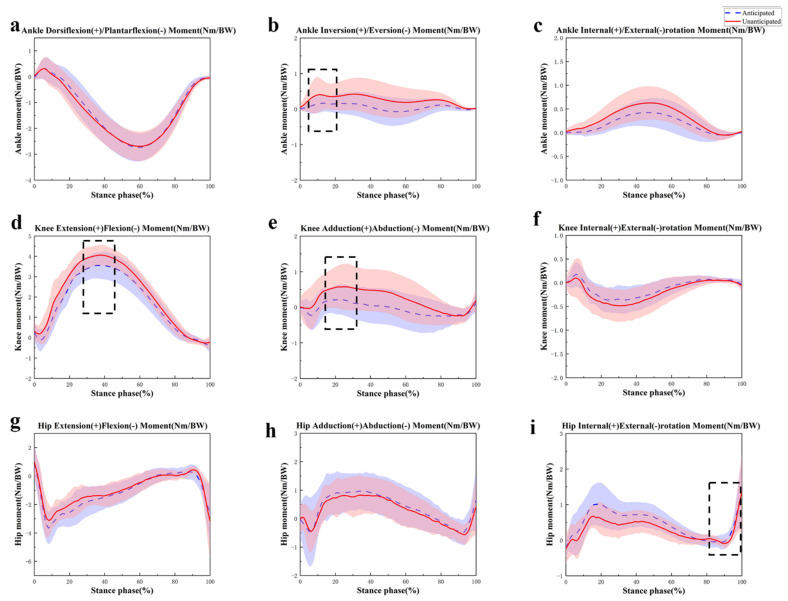
Curves of changes in the three-dimensional moments of sagittal ankle (**a**); frontal ankle (**b**); transverse ankle (**c**); sagittal knee (**d**); frontal knee (**e**); transverse knee (**f**); sagittal hip (**g**); frontal hip (**h**); and transverse hip (**i**) during 45° side-cutting under different conditions. Note: The dashed box index indicates a significant difference between the two side-cutting tasks with *p* < 0.05.

Table 4 presents the moments observed during 45° side-cutting tasks under different anticipation conditions. During the unanticipated task, there was a significantly larger peak ankle inversion (*p* < 0.001) and internal rotation (*p* < 0.001) moment compared to anticipated tasks (Figure 5b). However, the peak ankle eversion moment was significantly lower in unanticipated tasks (*p* < 0.001). There were no notable disparities in the maximum range of motion of the ankle joint in terms of dorsiflexion, plantarflexion, and external rotation moment.

For the knee joint, unanticipated 45° side-cutting tasks showed a significantly higher peak knee extension moment (*p* < 0.001), adduction moment (*p* < 0.001), and external rotation moment (*p* = 0.001) (Figure 5d,e). Conversely, the peak knee flexion moment was significantly lower during unanticipated tasks (*p* = 0.002). There was no significant difference in the peak knee abduction and internal rotation moments (Figure 5f).

At the hip joint, the peak flexion moment (*p* = 0.026) and external rotation moment (*p* = 0.028) were significantly larger during unanticipated 45° side-cutting tasks (Figure 5i). There were no significant differences between the two conditions in peak hip extension, adduction, abduction, and internal rotation moments (Figure 5g,h).

**Figure 6:**
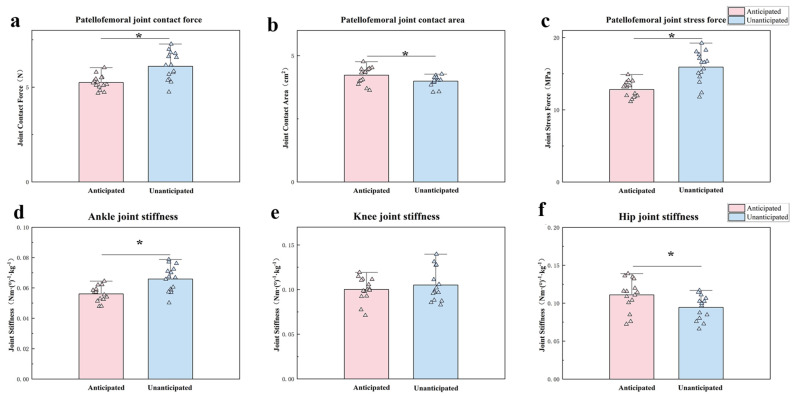
Comparison of patellofemoral joint contact force (**a**), contact area (**b**), stress force (**c**) ankle joint stiffness (**d**), knee joint stiffness (**e**), and hip joint stiffness (**f**) during anticipated and unanticipated tasks. The triangles represent the mean values for each participant. Note: The “*” represent significance with *p* < 0.05.

Table 5 displays the measurements of the patellofemoral joint contact force, contact area, and stress force, specifically during 45° side-cutting. The peak patellofemoral joint contact force (*p* = 0.022) and stress force (*p* = 0.011) were significantly greater during the unanticipated tasks (Figure 6a,c). Conversely, the peak patellofemoral joint contact area (*p* = 0.013) significantly decreased during the unanticipated 45° side-cutting (Figure 6b). The ankle joint stiffness was significantly greater than anticipated (*p* < 0.001). In contrast, the hip joint stiffness was significantly lower than anticipated (*p* = 0.002) (Figure 6d,f). Additionally, there was no significant difference in knee joint stiffness between the two conditions (Figure 6e).

**Figure 7:**
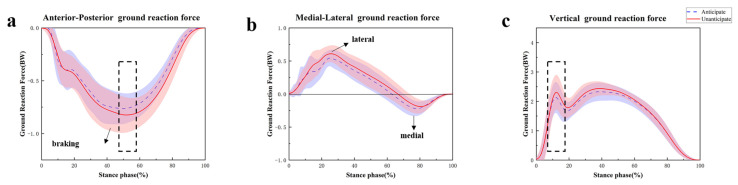
Curves of changes in the anterior and posterior ground reaction force (**a**), medial and lateral ground reaction force (**b**), and vertical ground reaction force (**c**) during 45° side-cutting under different conditions. Note: The black dashed line represents a significant difference between the two side-cutting tasks with *p* < 0.05.

The GRF findings during the different situations of 45° side-cutting are displayed in Table 6. The unanticipated side-cutting condition exhibited significantly greater peak braking (*p* = 0.034) and vertical force (*p* = 0.011) compared to the anticipated tasks (Figure 7a,c). Furthermore, no significant differences were observed in peak lateral and medial forces (Figure 7b).

**Figure 8:**
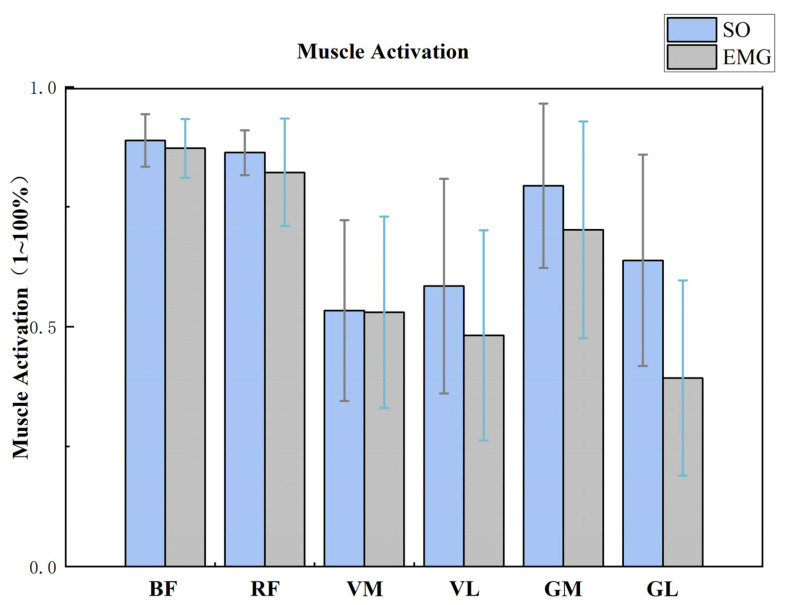
Comparison of estimated peak Opensim activation against EMG recorded signals. (BF: Biceps femoris, RF: Rectus femoris, VM: Vastus medialis, VL: Vastus lateralis, GM: Gastrocnemius medialis, GL: Gastrocnemius lateralis).

**Figure 9:**
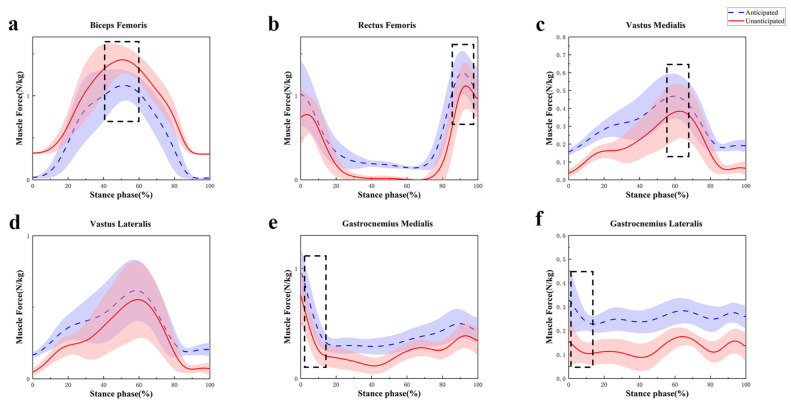
Curves of changes in the muscle force of biceps femoris (**a**), rectus femoris (**b**), vastus medialis (**c**), vastus lateralis (**d**), gastrocnemius medialis (**e**), and gastrocnemius lateralis (**f**) during anticipated and unanticipated tasks. Note: The black dashed line represents a significant difference between the two side-cutting tasks with *p* < 0.05.

The EMG activation variables were qualitatively compared with the muscle activations predicted by OpenSim simulations to assess the reliability of the OpenSim model (Figure 8). Figure 8 illustrates the comparison result, which indicates that the predicted muscle activation and EMG during the side-cutting tasks were in good agreement. Table 7 shows that during unanticipated side-cutting movements, the muscle forces of the rectus femoris (*p* < 0.001), vastus medialis (*p* < 0.001), lateral gastrocnemius (*p* < 0.001), and medial gastrocnemius (*p* < 0.001) all significantly decreased (Figure 9b,c,e,f). There was no significant difference in the biceps femoris and vastus lateralis muscle forces (Figure 9d). The muscle force of the biceps femoris was significantly increased (*p* < 0.001) during the unanticipated side-cutting task (Figure 9a).

## 4. Discussion

This study conducted a comparison of lower extremity joint angles, moments, stiffness, and contact forces in individuals with PFPS who engage in side-cutting exercises, both in planned and unforeseen activities. Compared to anticipated tasks, we found that the unanticipated side-cutting tasks have large knee joint peak abduction and internal rotation angles. This study also found that unanticipated tasks have large knee external rotation moments. The patellofemoral joint peak contact and stress force during the unanticipated task was higher than anticipated. Although the knee joint plays a vital function in the musculoskeletal system, there is still a lack of modelling for the patellar joint during side-cutting motions in both anticipated and unanticipated scenarios. Most existing studies have predominantly concentrated on the anterior cruciate ligament of the knee joint [17,19,20]. The impact of 45° side-cutting on the patellar joint is underexplored and warrants further investigation. This study aimed to examine the biomechanical mechanics of this particular action, providing valuable insights for both theoretical understanding and practical implementation. We used OpenSim simulations to explore the movement, forces, and muscle power of the kneecap and leg joints during a 45° sideways movement in persons with PFPS, both in expected and unexpected situations.

Regarding kinematics, we found that the knee joint exhibited larger valgus and internal rotation angles during the unanticipated 45° side-cutting task. Upon landing, the knee joint was initially positioned in a valgus condition, and the valgus angle was significantly larger than anticipated. Based on a prior investigation, the first motion at ground contact can only be accomplished by landing with the foot in eversion and tilting the torso outward [20,47]. The increased valgus and internal rotation angles of the knee affect sports performance and significantly heighten the risk of a non-contact knee injury [48,49,50,51]. Excessive knee valgus angles diminish the ability of the ligaments to limit movement in the knee joint, therefore raising the strain and likelihood of damage to the knee. Borotikar et al. found that the peak knee flexion angle increased by up to 50% during unanticipated side-cutting [52]. In contrast, our study did not observe an increase in knee flexion angle under unanticipated conditions. This difference may be attributed to the increased demand for knee stability during unanticipated tasks, where participants may reduce knee flexion to enhance stability and control in response to sudden changes in movement [18,53]. The results align with other studies and emphasize the need to effectively manage knee joint motion during side-cutting tasks to prevent injuries [54]. The results also indicate a significant increase in peak ankle dorsiflexion and plantarflexion angles during unanticipated tasks compared to anticipated tasks. This finding suggests that unanticipated movements demand greater adaptability in ankle control to maintain balance and directional change [55]. The reduction in peak inversion and eversion angles during unanticipated tasks further highlights the ankle’s role in stabilizing sudden directional changes. This aligns with previous research suggesting that the ankle joint role in compensatory strategies becomes more pronounced under unanticipated conditions [35]. Additionally, the increased ROM in the sagittal plane during unanticipated conditions indicates a more dynamic engagement of the ankle joint, which is crucial for reactive tasks [56]. When performing unanticipated side-cutting movements, consider using a muscle tape to maintain knee stability and reduce the joint’s range of motion.

The study’s kinetic results demonstrated that unexpected side-cutting led to an increased moment of knee adduction and external rotation, which is consistent with previous studies [57]. The knee joint’s requirement to properly handle the uneven load during an unexpected change in direction, especially during sideways motions, might explain this. This is achieved by raising the adduction and external rotation moments, which may increase the likelihood of the knee collapsing [18]. In addition, unanticipated 45° side-cutting greatly increases the maximum force and pressure on the PFJ while reducing the area of contact, as opposed to expected motions. The increase in contact force and stress force aligns with previous research, indicating that unanticipated tasks impose higher joint loads due to the need for rapid stabilization and force redistribution [58]. The reduction in contact area may further exacerbate the stress on the PFJ, as a smaller surface area bears greater force, potentially leading to a higher risk of joint degeneration or injury over time [59]. Unanticipated side-cutting demonstrated significantly higher peak braking and vertical forces, indicating that such tasks impose increased biomechanical demands on the lower extremities due to the abrupt requirements for deceleration and stabilization in response to unforeseen directional changes. The increased peak braking force aligns with prior studies indicating that unanticipated conditions require rapid force production for body control and stabilization. Similarly, the elevated vertical force suggests greater compressive loads on the lower extremities, likely due to the need to counteract inertia during sudden movement changes.

Related clinical practice has confirmed that quadriceps muscle strength abnormalities are also closely related to the onset of PFPS. In contrast, the unanticipated decrease in rectus femoris, medial latissimus, and gastrocnemius muscle strength is observed after a 45° side-cutting task [24]. Our observations coincide; however, we discovered that the proportional contributions of various muscle groups varied during side-cutting [60,61]. We discovered that unanticipated side-cutting imposes different demands on the musculature than anticipated movements. The decreased muscle forces observed in the RF, VM, and gastrocnemius muscles may indicate a reduced capacity for these muscles to stabilize the knee joint during unanticipated maneuvers [26,62,63]. This reduction in force could contribute to an increased risk of injury, as these muscles play crucial roles in knee extension and patellar stabilization [23]. The results suggest that athletes with PFPS should strengthen the quadriceps muscles, especially the biceps femoris and rectus femoris, in their later training.

This study has several limitations. Although the sample size was adequate for identifying differences, it was relatively small and consisted solely of male basketball players, which may limit the broader applicability of the findings. Including only male participants from a single location restricts the generalization of the results to female populations [30]. Given the previously established disparities in lower limb strength and biomechanics across genders, it is imperative to conduct more studies incorporating female subjects. Future studies should also examine these biomechanical responses in a more diverse athletic population and consider the long-term adaptations to unanticipated conditions.

## 5. Conclusions

This study investigated the biomechanical disparities between expected and unexpected side-cutting activities in athletes diagnosed with PFPS. The results demonstrate that unexpected motions substantially affect the movement patterns of the ankle, knee, and hip joints. These modifications indicate that unforeseen circumstances require more joint stability and adaptation, which might potentially raise the likelihood of damage. Training regimens that incorporate unexpected scenarios are necessary to enhance neuromuscular control and reactive agility because of the increased range of motion in ankle dorsiflexion and plantarflexion, as well as the altered mechanics of the knee and hip.

The findings also offer valuable insights for rehabilitation treatments in treating PFPS. Integrating activities that replicate unexpected movements can enhance joint stability and overall athletic performance. Subsequent investigations should prioritize a subject pool encompassing a more comprehensive range of diversity and incorporate supplementary biomechanical data to enhance the accuracy and precision of these findings. In summary, this study enhances our comprehension of the difficulties presented by unexpected movements in sports. It emphasizes the significance of focused therapies in reducing injury risks in PFPS athletes.

## Figures and Tables

**Table 1:** The variables of the ankle, knee, and hip angel during the 45° cutting tasks. (Mean ± SD).

Variables (°)		Anticipated	Unanticipated	*p*-Value	t
Peak ankle angle	Dorsiflexion	23.67 ± 5.10	25.28 ± 4.00	0.036 *	−2.141
	Plantarflexion	−25.59 ± 6.20	−32.02 ± 6.57	<0.001 *	5.633
	Inversion	−6.89 ± 4.76	−4.10 ± 5.09	0.003 *	−3.089
	Eversion	−22.51 ± 7.70	−17.41 ± 4.28	<0.001 *	−4.766
	Internal rotation	5.27 ± 2.52	5.37 ± 2.28	0.769	−0.295
	External rotation	−8.43 ± 3.81	−7.89 ± 2.82	0.386	−0.873
Peak knee angle	Flexion	−57.49 ± 6.37	−58.37 ± 5.40	0.349	−0.943
	Adduction	−5.50 ± 2.72	3.47 ± 1.86	<0.001 *	−4.812
	Abduction	−1.37 ± 3.35	−3.70 ± 2.77	<0.001 *	−6.200
	Internal rotation	7.14 ± 5.87	9.44 ± 5.27	0.003 *	3.094
	External rotation	−8.69 ± 4.19	−7.28 ± 4.06	0.052	1.984
Peak hip angle	Flexion	−14.63 ± 7.31	−15.75 ± 7.85	0.275	4.897
	Adduction	18.15 ± 4.52	15.70 ± 3.81	<0.001 *	3.619
	Abduction	−0.59 ± 4.01	−2.69 ± 3.04	0.005 *	2.911
	Internal rotation	6.93 ± 6.37	5.42 ± 3.21	0.073	1.823
	External rotation	−8.78 ± 5.09	−9.04 ± 3.03	0.686	0.406

Note: The bold and “*” represents significance with *p* < 0.05.

**Table 2:** The ROM of the ankle, knee, and hip angel during the 45° cutting tasks. (Mean ± SD).

Variables (°)		Anticipated	Unanticipated	*p*-Value	t
ROM of ankle	Sagittal plane	49.26 ± 8.17	57.30 ± 7.24	<0.001 *	−5.470
	Frontal plane	15.62 ± 5.70	13.31 ± 3.89	0.008 *	2.743
	Transverse plane	13.70 ± 3.65	13.26 ± 2.96	0.481	0.708
ROM of knee	Sagittal plane	43.68 ± 5.60	45.43 ± 6.68	0.112	1.161
	Frontal plane	6.86 ± 1.84	7.44 ± 2.32	0.069	1.851
	Transverse plane	15.82 ± 4.28	16.72 ± 3.65	0.126	1.553
ROM of hip	Sagittal plane	62.16 ± 8.33	59.23 ± 10.30	0.023 *	2.337
	Frontal plane	18.74 ± 5.04	18.39 ± 5.08	0.591	0.540
	Transverse plane	15.17 ± 5.42	14.46 ± 3.67	0.144	1.481

Note: The bold and “*” represents significance with *p* < 0.05.

**Table 3:** The initial foot contact angle of ankle, knee, and hip during 45° side-cutting. (Mean ± SD).

Variables		Anticipated	Unanticipated	*p*-Value	t
Ankle	Dorsiflexion/Plantarflexion (°)	7.47 ± 4.89	7.57 ± 3.85	0.914	−0.109
	Inversion/Eversion (°)	−9.06 ± 5.74	−6.53 ± 5.11	0.011 *	−2.612
	Internal/External rotation (°)	−0.29 ± 3.05	−0.34 ± 3.02	0.921	0.100
Knee	Flexion/Extension (°)	−24.80 ± 6.93	−27.15 ± 6.76	0.094	−1.700
	Adduction/Abduction (°)	−0.75 ± 3.10	−2.19 ± 2.36	<0.001 *	−3.816
	Internal/External rotation (°)	4.29 ± 6.06	6.00 ± 4.09	0.041	2.089
Hip	Flexion/Extension (°)	47.53 ± 5.47	43.01 ± 4.63	<0.001 *	3.593
	Adduction/Abduction (°)	2.74 ± 4.10	−0.34 ± 3.46	<0.001 *	5.013
	Internal/External rotation (°)	3.39 ± 4.78	−4.31 ± 4.30	0.134	1.518

Note: The bold and “*” represents significance with *p* < 0.05.

**Table 4:** The variables of the ankle, knee, and hip moment during the 45° cutting tasks. (Mean ± SD).

Variables		Anticipated	Unanticipated	*p*-Value	t
Peak ankle moment	Dorsiflexion (Nm/BW)	0.43 ± 0.42	0.43 ± 0.39	0.883	0.148
	Plantarflexion (Nm/BW)	−2.76 ± 0.53	−2.72 ± 0.55	0.557	0.590
	Inversion (Nm/BW)	0.31 ± 0.22	0.61 ± 0.39	<0.001 *	6.412
	Eversion (Nm/BW)	−0.25 ± 0.23	−0.14 ± 0.21	<0.001 *	3.559
	Internal rotation (Nm/BW)	0.48 ± 0.27	0.66 ± 0.33	<0.001 *	3.799
	External rotation (Nm/BW)	−0.12 ± 0.09	−0.10 ± 0.09	0.360	0.923
Peak knee moment	Extension (Nm/BW)	3.66 ± 0.63	4.17 ± 0.54	<0.001 *	4.383
	Flexion (Nm/BW)	−0.67 ± 0.33	−0.50 ± 0.23	0.002 *	3.266
	Adduction (Nm/BW)	0.53 ± 0.30	0.86 ± 0.48	<0.001 *	4.963
	Abduction (Nm/BW)	−0.57 ± 0.35	−0.59 ± 0.46	0.707	−0.377
	Internal rotation (Nm/BW)	0.31 ± 0.22	0.31 ± 0.38	0.946	−0.068
	External rotation (Nm/BW)	−0.47 ± 0.26	−0.91 ± 0.33	0.001 *	−3.376
Peak. hip moment	Extension (Nm/BW)	1.28 ± 0.64	1.46 ± 0.76	0.160	−1.424
	Flexion (Nm/BW)	−4.49 ± 0.88	−4.90 ± 0.98	0.026 *	2.276
	Adduction (Nm/BW)	1.53 ± 0.77	1.46 ± 0.62	0.543	0.612
	Abduction (Nm/BW)	−1.24 ± 1.10	−1.01 ± 0.62	0.087	−1.743
	Internal rotation (Nm/BW)	1.66 ± 0.60	1.73 ± 0.63	0.563	−0.592
	External rotation (Nm/BW)	−0.47 ± 0.25	−0.59 ± 0.42	0.028 *	2.259

Note: The bold and “*” represents significance with *p* < 0.05.

**Table 5:** The patellofemoral joint contact force, contact area, stress force, and joint stiffness of the ankle, knee, and hip during cutting. (Mean ± SD).

Parameters	Anticipated	Unanticipated	*p*-Value
Peak PTF (N)	5.25 ± 1.41	6.10 ± 2.12	0.022 *
Peak PFCA (cm^2^)	4.25 ± 0.46	4.00 ± 0.50	0.013 *
Peak PP (MPa)	12.83 ± 4.52	15.93 ± 7.20	0.011 *
Ankle stiffness (Nm·(°)^−1^·kg^−1^)	0.06 ± 0.01	0.07 ± 0.01	<0.001 *
Knee stiffness (Nm·(°)^−1^·kg^−1^)	0.10 ± 0.02	0.11 ± 0.02	0.214
Hip stiffness (Nm·(°)^−1^·kg^−1^)	0.11 ± 0.03	0.09 ± 0.02	0.002 *

Note: The bold and “*” represents significance with *p* < 0.05.

**Table 6:** The ground reaction force during 45° side-cutting. (Mean ± SD).

Parameters	Anticipated	Unanticipated	*p*-Value
Peak Breaking Force (BW)	−0.79 ± 0.14	−0.84 ± 0.16	0.034 *
Peak Lateral Force (BW)	0.64 ± 0.21	0.68 ± 0.13	0.173
Peak Medial Force (BW)	−0.23 ± 0.10	−0.20 ± 0.09	0.073
Peak Vertical Force (BW)	2.59 ± 0.30	2.72 ± 0.38	0.011 *

Note: The bold and “*” represents significance with *p* < 0.05.

**Table 7:** The muscle force in side-cutting movements during anticipated and unanticipated tasks (Mean ± SD).

Parameters	Anticipated	Unanticipated	*p*-Value
Biceps femoris (BF)	1.19 ± 0.22	1.49 ± 0.18	<0.001 *
Rectus femoris (RF)	1.36 ± 0.26	1.17 ± 0.26	<0.001 *
Vastus medialis (VM)	0.50 ± 0.14	0.42 ± 0.15	<0.001 *
Vastus lateralis (VL)	0.67 ± 0.24	0.63 ± 0.25	0.075
Gastrocnemius medialis (GM)	0.96 ± 0.21	0.76 ± 0.24	<0.001 *
Gastrocnemius lateralis (GL)	0.38 ± 0.08	0.24 ± 0.05	<0.001 *

Note: The bold and “*” represents significance with *p* < 0.05.

## Data Availability

The data that support the findings of this study are available on reasonable request from the corresponding author.
